# Factors associated with prescription of oral anticoagulants in the era of direct oral anticoagulants

**DOI:** 10.1002/clc.24100

**Published:** 2023-08-15

**Authors:** Naoya Kataoka, Teruhiko Imamura

**Affiliations:** ^1^ Second Department of Internal Medicine University of Toyama Toyama Japan

To the Editor,

Appropriate prescription of oral anticoagulants (OACs) for patients with atrial fibrillation (AF) and high stroke risk is of great importance. Manning^1^ and colleagues evaluated the prevalence of OAC prescription within 6 months after the first diagnosis of AF in patients at high stroke risk during the observation period between 2013 and 2018.^1^ They demonstrated that most newly diagnosed AF patients at high stroke risk did not receive OAC prescription within the first 6 months after the diagnosis. Several parameters were associated with the prescription/nonprescription of OACs.

The total prescription rate of OACs, as well as that of direct OACs (DOACs), increased yearly during the observation period.^1^ Now is the era of DOAC,^2^ and it may be ideal to start the study period from the time when DOAC came in the market. Could the authors explain the reason why they defined the study period between 2013 and 2018?

In another large database study, 70% of AF patients received OACs.^3^ Patients included in the authors’ study may have had relatively lower CHA2DS2‐VASc score to receive OACs. Could the authors present participants’ CHA2DS2‐VASc score to clarify the clinical indication of OAC therapy?

In the authors’ study, the use of antiarrhythmic drugs was associated with a lower rate of OAC prescription, which is inconsistent with other previous studies.^1^ This is probably due to the old concept: rhythm control versus rate control, in which rhythm control is sufficient to prevent stroke without anticoagulation.^2^ Again, the observation period may be relatively too old to apply their findings to the current era.

Recently, left atrial appendage closure is indicated if it is challenging to continue OACs in patients with AF and high stroke risk.^4^ In their study, some patients who did not receive OACs despite its indication may have received left atrial appendage closure, which prevents stroke events without OACs. Could the authors show the prevalence of patients receiving left atrial appendage closure?

## CONFLICT OF INTEREST STATEMENT

The authors declare no conflicts of interest.
